# Supplementary material for: Identification of photoperiod-induced specific miRNAs in the adrenal glands of Sunite sheep (Ovis aries)
Source: Front Vet Sci. 2022 Jul 22;9:888207. doi: 10.3389/fvets.2022.888207 (PMC9354845; doi:10.3389/fvets.2022.888207)
Supplement: Supplementary file 1 [file Data_Sheet_1.ZIP › Supplementary file/miRDeep2 file.zip!/miRDeep2 file/Know_Structure/LP42A2/oar-mir-25.pdf]

miRBase precursor	: oar-mir-25
Total read count	: 16039
oar-miR-25 read count	: 15966
remaining reads	: 73

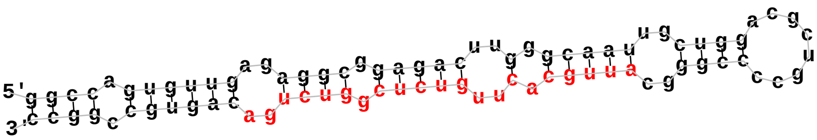

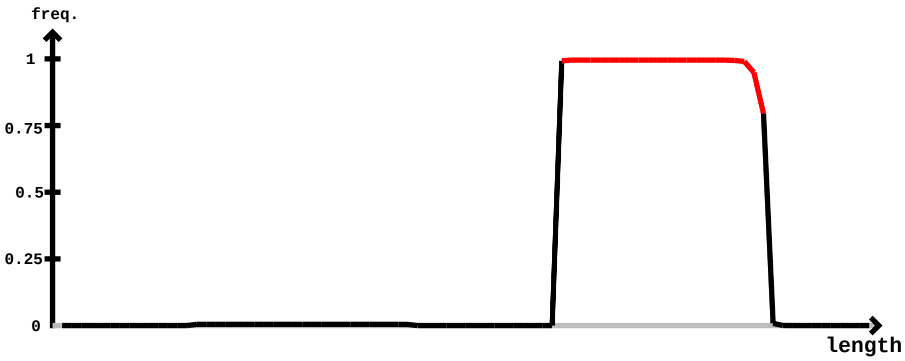

oar-miR-25

```
5'- ggccaguguugagaggcggagacuugggcaauugcuggacgcugccccgggcauugcacuugucucggucugacagugccggcc   -3'    exp
    ((((.((((((..((((.(((((..(.(((((..((((........))))..))))).)..))))).)))).)))))).))))   reads      mm         sample
    .............aggcggagacuugggcaau....................................................   1          0          seq
    .............aggcggagacuugggcaauug..................................................   2          0          seq
    .............aggcggagacuugggcaauugc.................................................   2          0          seq
    .............aggcggagacuugggcaauugcu................................................   68         0          seq
    ..................................................gcauugcacuugucucggucuga...........   2          0          seq
    ...................................................cauugcacuugucucgg................   3          0          seq
    ...................................................cauugcacuugucucggu...............   18         0          seq
    ...................................................cauugcacuugucucgguc..............   62         0          seq
    ...................................................cauugcacuugucucggucu.............   656        0          seq
    ...................................................cauugcacuugucucggucug............   2468       0          seq
    ...................................................cauugcacuugucucggucuga...........   12579      0          seq
    ...................................................cauugcacuugucucggucugac..........   129        0          seq
    ...................................................cauugcacuugucucggucugaca.........   5          0          seq
    ....................................................auugcacuugucucggucu.............   3          0          seq
    ....................................................auugcacuugucucggucug............   4          0          seq
    ....................................................auugcacuugucucggucuga...........   37         0          seq
```
